# Giant Intraabdominal Lymphangioma in a Pediatric Patient—A Challenging Diagnosis

**DOI:** 10.3390/clinpract14030059

**Published:** 2024-04-25

**Authors:** Iuliana-Laura Candussi, Alexandru Petecariu, Mirela Lungu, Camelia Busila, Raul Mihailov, Anca Neagu, Claudiu N. Lungu, Ioan Sarbu, Carmen I. Ciongradi

**Affiliations:** 1 Department of Pediatric Surgery, Clinical Country Children Emergency Hospital, Faculty of Medicine and Pharmacy, ‘Dunarea de Jos’ University, 800010 Galati, Romania; iuliana.candussi@ugal.ro (I.-L.C.); alexpetecariu@yahoo.com (A.P.); mirelacrainiciuc@gmail.com (M.L.); camelia_busila@yahoo.com (C.B.); 2 Department of Surgery, Clinical Country Emergency Hospital, 800578 Galati, Romania; raul.mihailov@ugal.ro; 3 Department of Pathology, Clinical Country Children Emergency Hospital, Faculty of Medicine and Pharmacy, ‘Dunarea de Jos’ University, 800010 Galati, Romania; ancazanoschi@gmail.com; 4 Department of Functional and Morphological Science, Faculty of Medicine and Pharmacy, ‘Dunarea de Jos’ University, 800010 Galati, Romania; 5 2nd Department of Surgery—Pediatric Surgery and Orthopedics, “Grigore T. Popa” University of Medicine and Pharmacy, 700115 Iași, Romania; carmen.ciongradi@umfiasi.ro

**Keywords:** intra-abdominal cyst, large masses, mesenteric lymphangioma

## Abstract

**Introduction**: Intra-abdominal cystic formations represent heterogeneous pathologies with varied localization and clinical manifestation. The first challenge of a giant intra-abdominal cystic lesion is identifying the organ of origin. The clinical presentation of intra-abdominal cystic lesions varies from acute manifestations to non-specific symptoms or accidental discovery. **Case presentation**: A 2-year-old girl presents to the emergency unit with a fever of 38.5 Celsius, loss of appetite, and apathy. The investigations showed a gigantic intra-abdominal mass whose organ belonging could not be specified. Postoperatively, a giant mesenteric lymphangioma was evident, which was completely excised. **Discussion**: Giant cystic formations modify the anatomical reports and become space-replacing formations, and the starting point is even more challenging to assess preoperatively. Nevertheless, the careful evaluation of the characteristics of the formation, the effect on the adjacent organs, the age of the patient, and the clinical picture can provide elements of differential diagnosis. The stated purpose of this work is to systematize intra-abdominal lesions according to the organ of origin and to make the preoperative diagnosis of an intra-abdominal cystic lesion in the pediatric patient easy to perform starting from the presented case.

## 1. Introduction

Intra-abdominal cystic formations represent heterogeneous pathologies with varied localization and clinical manifestation. The first challenge in front of a giant intra-abdominal cystic lesion is identifying the starting organ [1,2]. The clinical picture of intra-abdominal cystic lesions varies from acute manifestations, non-specific symptoms, or accidental discovery [3,4]. The mass effect in giant formations causes non-specific symptomatology, which can delay the diagnosis. This phenomenon also happened in the presented case. The association of information obtained from imaging investigations [ultrasound, C.T., M.R.I.], laboratory analyses, and the clinical picture is essential to obtaining a preoperative diagnosis. However, the therapeutic strategy can be changed intraoperatively, depending on the characteristics of the lesion [5,6,7]. The purpose of this work is that starting from the presented case, we tried to systematize intra-abdominal lesions according to the organ of origin and to make the preoperative diagnosis of an intra-abdominal cystic lesion in the pediatric patient easy to perform. The preoperative identification of the characteristics of an intra-abdominal formation and the effect on the adjacent organs facilitate surgical intervention and shorten the anesthetic time. 

## 2. Case Presentation

A 2-year-old girl presented to the emergency service with a fever of 38.5 °C, loss of appetite, and apathy. The febrile syndrome had started 2–3 days previously. The administration of NSAIDs slightly improved it, and following the pediatric consultation, the diagnosis was a mild respiratory infection. However, the apathy and lack of appetite were present for approximately 4–5 months; they evolved progressively and were added to transit disorders and weight stagnation. The medical file was incomplete due to the family’s lack of involvement, negatively impacting diagnostic establishment and overall medical decisions. Also, the girl was placed in the care of the state, which resulted in a delayed transfer of medical information and consecutively to a delay in the diagnosis. The child has been institutionalized for about one year, so there is no data on her health status from the first year of life. For about a week, the patient refused food, was apathetic, had reduced motor activity, refused to mobilize, and preferred static activities. She became agitated when placed supine, preferring the lateral position. The clinical examination noted that the abdomen had an increased globular volume without collateral circulation and a decrease in fatty tissue on the abdomen, chest, limbs, and face. She did not present with palpable adenopathies. At presentation, the patient weighed 11 kg, and I.P. was 0.65. On palpation, the abdomen was slightly depressed, without muscular contracture; palpating an abdominal tumor with liquidity character, which occupies the entire abdominal cavity, could be mobilized during bimanual palpation.

An abdominal ultrasound highlighted a voluminous abdominopelvic cystic expansive formation with an irregular outline delimited by a thin wall. The lesions were multiloculated and multiseptated, with several solid hyperechoic areas adhering to the septa. The axial dimensions of the lesions were 160/90 mm; the described lesion moved the intestinal loops and both kidneys, with the demarcation line present posteriorly. The uterus and left ovary did not have modified echographic characters, and intraperitoneal fluid was absent.

To accurately specify the diagnosis, an abdominal pelvic M.R.I. was performed. It showed a voluminous abdominal-pelvic cystic expansive formation, with a convex contour, relatively well delimited by a thin wall, multiloculated, and multiseptate, with moderate parietal gadolinophilia, and at the level of the septa, partially altered content due to the presence of areas with intermediate T2 signal, with dimensions of approximately 86/157/230 mm [AP/T/CC]. The described formation presents the following reports:

- Superior, the lesion has a mass effect on the liver, spleen, stomach, and transverse colon, which appeared to be displaced superiorly, with the border of separation;
- Inferior, the lesion comes in contact with the upper wall of the urinary bladder, with the limit of separation;
- Posteriorly, the lesion comes into contact with the intestinal loops
- Anteriorly, it comes in contact with the anterior abdominal wall

**Figure 1:**
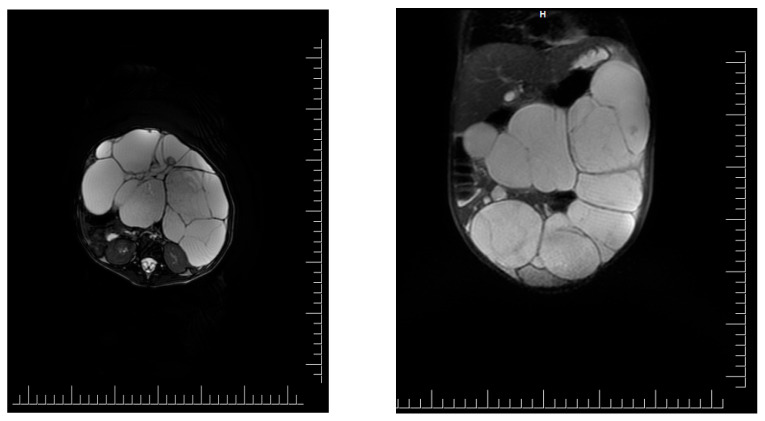
MRI scan of intrabdominal mass. Cystic masses in close relation to intrabdominal structures are observed. However, on an M.R.I. scan, no involvement of the intrabdominal structures is observed.

Ovaries are challenging to visualize, imprinted by the formation described—the uterus without expansive formations. The urinary bladder was examined in fullness, regular parietal contour, and homogeneous liquid. The liver had an anteroposterior diameter of the right lobe of ~117 mm and the lobe left of ~65 mm, with a homogeneous structure without focal lesions. There were undilated intra-/extrahepatic bile ducts. The cholecyst was dilated and had a wall with average thickness and liquid content, without stones. The spleen had a 74 mm diameter and a homogeneous structure. Pancreas, adrenal glands, and both kidneys had normal M.R.I. appearance. Intraperitoneal liquid had a maximum thickness at the pelvic level of 10 mm. There was no abdominal and pelvic adenopathy. No suspicious bone lesions were noted (Figure 1).

Laboratory tests revealed WBC 22,000 × 10^3^/mm^3^, RBC 3.73 × 10^6^/uL, hemoglobin 8.99 g/dL, hematocrit 28%, C reactive protein 7.77 mg/dL, total proteins 4.8 g/dL, V.S.H. 92 mm/h, and procalcitonin 3.9 ng/mL. The preoperative preparation of the patient aimed to correct the imbalances by administration of Albumin, Vitalipid, enteral solutions of electrolytes, and energetically enriched enteral solutions. 

**Figure 2:**
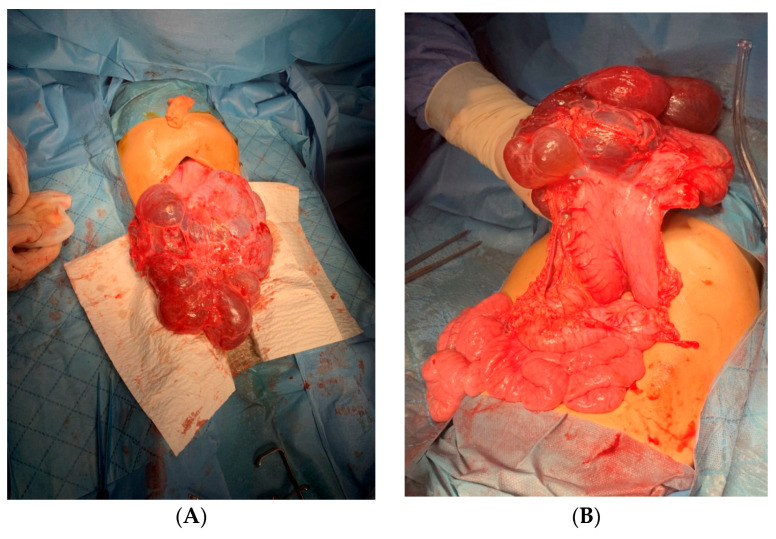
Intraoperative view of the peritoneal mass (**A**): general view; (**B**) the attachment with the surrounding structures is demonstrated. As observed on the M.R.I. scan, the intrabdominal masses were well defined, with no extra vascularization and no involvement of the nearby structures.

Surgical intervention was decided, and median exploratory laparotomy was performed; the abdominal formation tends to herniation when entering the peritoneal cavity. The cyst had adhesion to the peritoneum. It was multilobed and well defined, with a thin wall through the transparency. Adhesions were lysed by digitoclasia and electrocautery. We made a breach in the cystic cavity, which decompressed and exteriorized. The tumor had a diameter of 22/15 cm after partial decompression and started from the great omentum. It had a contact area with greater curvature of the stomach, and all the small intestinal loops were agglutinated subhepatically. The tumor was excised entirely [Figure 2], an intraperitoneal drain was placed, and the abdominal wall was closed.

The subsequent evolution was favorable; with the resumption of intestinal transit in 24 h, the intraperitoneal drain was removed on the fourth postoperative day. The patient remained hospitalized for 14 days, and during this period, the nutritional recovery was initiated so that in 2 months, the patient gained 1400 g in weight. Also, appetite and intestinal transit normalized. In addition, motor activity has improved, as has the patient’s interest in age-specific activities. The patient was clinically and sonographically monitored at three months. The serial ultrasounds performed did not show the recurrence of the lesions. 

**Figure 3:**
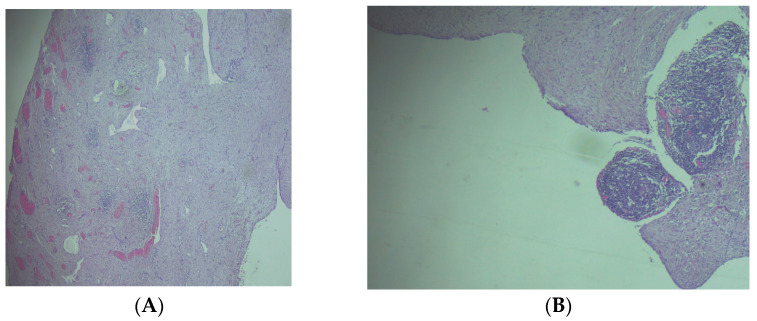
(**A**) The histological aspect of the cystic wall—consisted of connective stroma, with variable-sized vascular spaces and small lymphoid aggregates, H&E stain, magnification 40×. (**B**) The cysts were lined by a single layer of flattened endothelial cells, and there were peripheral lymphoid aggregates in the stroma, H&E stain, and magnification 100×.

**Figure 4:**
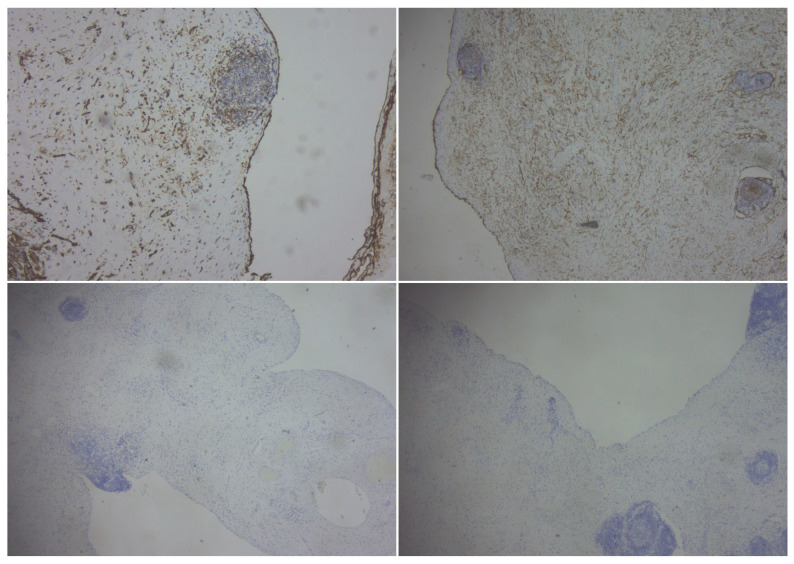
Immunohistochemical findings. In the upper left, CD31 [at magnification 100×], and in the upper right, Podoplanin [40× magnification], were positive in the lining of the cystic walls. Calretinin and PAX8 [40× magnification] were harmful in the lower quadrants.

The gross examination of the resection specimen revealed a multicystic mass with a smooth external surface, measuring 17 × 14 × 4 cm, filled with serous type fluid and a soft inner lining. The walls of the cysts had variable widths, a grey-pink color, and elastic consistency. At the microscopic examination, the walls of the cysts were lined by flattened, bland cells and consisted of varying amounts of fibro collagenous stroma with lymphoid aggregates. Immunohistochemistry was performed, and the lining cells were positive for CD31 and Podoplanin and negative for WT1 and PAX8, confirming the diagnosis of cystic lymphangioma (Figure 3 and Figure 4). 

## 3. Discussion

In total, 90% of lymphangiomas occur in children under two years of age. They are preferentially located in the head and neck [75%] and axilla [20%]. The abdominal location of lymphangiomas is rare, and precisely, this location allows the lesion to grow and cause late symptoms [8]. Intra-abdominal lymphangioma is a rare entity. In the abdomen, lymphangioma occurs most commonly in the mesentery, followed by the omentum, mesocolon, and retroperitoneum. After reaching a diameter of 13 cm, the cystic lesion might have become symptomatic by compression or lateral displacement of intestinal loops.

Abdominal lymphangiomas are classified as follows [9]:

- I—pedicled with the clinical manifestation dominated by torsion;
- II—sessile located between the layers of the mesentery;
- III—retroperitoneal;
- IV—multicentric with the involvement of both intraperitoneal and retroperitoneal organs.

The starting point of an intra-abdominal cystic lesion can be a parenchymal organ. The organ can be identified quickly in these conditions, allowing the doctor to focus on the differential diagnosis of organ lesions. However, with increased mobility, the cysts that develop from the rest of the intra-abdominal organs have centrifugal development and occupy the same space, the abdominal cavity [10,11,12]. This determines that these lesions have similar characteristics, making it challenging to identify the organ belonging before the operation. Giant cystic formations modify the anatomical reports and become space-replacing formations, and the starting point is even more difficult to assess preoperatively. Ultrasonography, computed tomography, and magnetic resonance imaging studies are helpful for diagnosis and surgical planning of abdominal cyst lymphangioma by determining its location and relation to surrounding structures [7,13]. Nevertheless, the careful evaluation of the characteristics of the formation, the effect on the adjacent organs, the age of the patient, and the clinical picture can provide elements of differential diagnosis [14]. To characterize a cystic lesion from an imagistic view, the following elements must be observed: the contents of the cyst, the unilocular or multilocular type, the thickness of the walls, the presence of internal septa, the presence of calcifications, the presence of a solid component, debris, or blood. In Table 1, the characteristics of formations starting from an intra-abdominal organ are given [1,4,5,15].

Abdominal cystic lesions have different symptomatology depending on the organ of origin and size. However, when the lesions are extensive and become space-replacing formations, the present symptomatology is non-specific, determined by their mechanical complications [13,14].

The clinical picture varies from incidental discovery to acute abdomen. The most common clinical form is non-specific symptomatology of partial intestinal obstruction, anorexia with a palpable, mobile, recalcitrant, painless tumor mass, nausea, and stagnation/loss of weight. Children can also present recurrent intestinal transit disorders [16,17,18].

Although laparoscopic treatment is an elegant method, it was not an option due to the voluminous lesion. As cited in the specialized literature, laparoscopic techniques for the excision of voluminous lesions presuppose punction of the lesion, with partial decompression to obtain the space necessary for surgical maneuvers. Laparoscopic treatment of voluminous intra-abdominal lesions is challenging due to the increased risk of injuring the lesion, the impossibility of obtaining a suitable working space, and the risk of incompletely excising the lesion. Sclerotherapy is a form of palliative treatment with limited indications for children due to local complications and the high rate of recurrence. The main indication for sclerotherapy is the proximity of a major blood vessel, which makes surgical resection risky [19].

The presented patient had non-specific symptoms, and her visit to the doctor was due to a respiratory infection. Lymphangioma is a benign congenital lesion that occurs due to the failure of communication in the intrauterine period [20] between the small intestinal lymphatic vessels and the major intestinal lymphatic vessels, which causes the cystic dilation of the lymphatic space [21,22]. The incidence of lymphangioma of the omentum is low, with approximately 1:20,000 hospitalizations in pediatric hospitals, most under the age of 10 and with an average age of appearance of 4.5 years [23,24]. The condition is slightly more common in males in the pediatric population [60%] than in the adult population [predominantly in women]. Mesenteric lymphatic cysts are four times more common than omental cysts [25,26]. In the presented patient, the location was at the level of the greater omentum. The localization at the level of the omentum allows an insidious development of the lesion, with the appearance of symptoms, the diameter of the lesion exceeds 13 cm. However, the literature also describes locations such as lesions at the level of the gastrocolic or gastro-lineal ligament [27,28]. Therefore, surgical treatment with a curative visa is the treatment of choice. In the presented case, this objective was accomplished without recurrence postoperatively in the first six months, significantly improving the quality of life [29].

Preoperative diagnosis of intra-abdominal cystic lesions usually requires imaging investigations, clinical examination, and maybe lab testing. Several standards could be taken into account, including the clinical symptoms. Individuals may display discomfort, distension, abdominal pain, or a palpable mass. These symptoms’ nature, strength, and length can offer vital insights. Imaging studies are also critical. Ultrasound is one of the imaging modalities used to assess intra-abdominal cystic lesions. As the first imaging modality is non-invasive, it is often used. It can disclose the cystic lesion’s characteristics, location, and size. Computed tomography, or C.T. scan, provides highly detailed cross-sectional images of the abdomen, making it easier to characterize lesions more precisely, assess how the lesion interacts with surrounding tissues, and look for any features that might point to cancer. M.R.I., or magnetic resonance imaging, aids in evaluating soft tissue architecture and separating solid masses from cystic lesions. It might provide more information about the kind and makeup of the cyst. X-rays are good as a first screening tool but not as informative as other imaging modalities. Furthermore, laboratory tests are essential. Although blood tests are not often used as a stand-alone diagnostic tool for intra-abdominal cystic lesions, they may be ordered if malignancy is suspected to look for indications of inflammation, infection, or tumors. The characteristics of the lesion are evaluated based on imaging studies—factors such as location, size, form, wall thickness, internal content (fluid density, solid component presence, or septation existence), and the presence of calcifications are evaluated. The clinical history and risk factors play a crucial role. The patient’s medical history, including any prior surgeries, trauma history, and family history of cystic illnesses or malignancies, is considered along with pertinent risk factors, such as exposure to carcinogens. Finally, fine-needle aspiration (F.N.A.) or biopsy are to be considered. Depending on the situation, a biopsy or F.N.A. may be required to extract a sample of the cystic lesion for pathological analysis. This is especially true if a malignancy is suspected or the diagnosis is still uncertain after imaging tests. A commonly used algorithm is based on imaging results and clinical judgment. This algorithm, which is a simplified form, provides a general framework and can be tailored to specific patient characteristics, institutional policies, and resource availability. Overall, clinical judgment and experience are necessary to identify and treat intra-abdominal cystic lesions (Table 2) [1,30,31,32].

The therapeutic option is determined by each patient’s clinical presentation, illness features, and past treatment history. To customize the treatment plan for each patient, a multidisciplinary approach combining surgeons, oncologists, hematopathologists, and other specialists is frequently required.

## 4. Conclusions

Intra-abdominal cystic lesions represent a challenge for both the radiologist and the surgeon. The investigations cannot accurately suggest the type of injury, so the therapeutic plan can change intraoperatively. In the presented case, the lesion was proven postoperatively to have a starting point in the greater omentum and could be completely resected. Overall, a multidisciplinary team is often necessary to diagnose and treat these lesions accurately. Imaging studies play a central part in the diagnosis. Also, an algorithmic approach is crucial in evaluating and treating these lesions.

## Figures and Tables

**Table 1:** Abdominal cystic lesion and related symptomatology concerning organ topology.

Organ	Lesion	Characteristics
Liver and biliary tract	Hydatid cyst	Single, circular cystic lesions located in the liver parenchyma
	Mesenchymal hamartoma	Septate lesions, starting from the right lobe of the liver, can reach large dimensions with a compressive effect
	Hemangioma	The single, circular lesion, located in the liver parenchyma, with variable sizes
Spleen	Splenic cyst	Uniloculated oval/circular lesion located in the splenic parenchyma with dimensions of 6–7 cm
Pancreas	Pancreatic cyst	Uniloculated lesion, placed at the level of the tail/pancreatic body, reaches dimensions of 4–5 cm.
Kidney	Hydronephrosis	Cystic dilatation of the renal calyces and the renal pelvis with the reduction of the renal parenchyma
	Polycystic kidney disease	Round/oval cystic lesion with a retroperitoneal starting point with the absence of renal parenchyma
	Mesenteric cyst	Uni/multiloculated cystic lesion with a thin wall may have a retroperitoneal extension
Gastro-intestinal	Epiploic cyst	Uni/multiloculated cystic lesions can reach large sizes with calcifications or intralesional debris
	Enteric duplication cyst	They are tubular or globular, have a thick wall, have no septa, and the common wall is highlighted
	Ovarian cyst	Uniloculated or multiloculated lesions, pelvic starting point, and can reach large sizes
	Urachal cyst	The single cystic lesion, located on the midline, has a thick wall that imprints the urinary bladder
Genito—urinary	Ovarian teratomas	Cystic formation with an average diameter of 10 cm, with mixed liquid/solid content and intracystic calcifications
	Intra-abdominal abscess	Cystic lesion with thick wall, with infiltration of adjacent tissues, with nonhomogeneous content
Other injuries	Cystic teratomas	Uniloculated cystic lesion, with nonhomogeneous content with a pelvic starting point

**Table 2:** Algorithm steps in diagnostic of pediatric intra-abdominal cystic lesions (#—number).

#	Step	Description	Ref
1	Clinical Evaluation	Examine the patient’s medical history, considering the symptoms, length of time, and any pertinent conditions. Conduct a physical examination, using a feeling around the abdomen to check for lumps or sore spots.	[33]
2	Initial Imaging Evaluation	Ultrasound should be the first imaging modality used. Examine the cystic lesion’s location, size, and features. If more imaging modalities are required, consider the ultrasound results.	[34]
3	Further Imaging Evaluation	If the ultrasound results are unclear or more characterization is required, move on to a CT scan or MRSI. A CT scan offers comprehensive pictures of the abdomen and aids in determining how the cystic lesion interacts with surrounding structures. M.R.I. can give more details regarding the cyst’s contents and is helpful in characterizing soft tissues.	[35]
4	Characterization of the Lesion	Determine the cystic lesion’s size, location, shape, wall thickness, internal content, and existence of septations or solid components based on the imaging results.	[36]
5	Consider Differential Diagnosis	Based on imaging features and clinical presentation, examine several differential diagnoses, such as benign cysts (simple cysts and pseudocysts) and malignant lesions (such as cystic tumors and cystic metastases).	[37]
6	Pathological evaluation (if necessary)	In cases where a malignancy is suspected or the diagnosis is still ambiguous following imaging examinations, consider doing a biopsy or fine-needle aspiration (F.N.A). The sample can be pathologically examined to learn more about the type of lesion and to help with treatment planning.	[38]
7	Multidisciplinary Review	Reviewing imaging results, pathology findings, and clinical presentation may require consultation with radiologists, surgeons, and pathologists. After discussing the diagnosis, the multidisciplinary team develops a management strategy based on their combined experience.	[39]

## Data Availability

On reasonable demand.
